# One-month assessment of Th-cell axis related inflammatory cytokines, IL-17 and IL-22 and their role in alcohol-associated liver disease

**DOI:** 10.3389/fimmu.2023.1202267

**Published:** 2023-12-14

**Authors:** Manasa Sagaram, Jane Frimodig, Danielle Jayanty, Huirong Hu, Amor J. Royer, Ryne Bruner, Maiying Kong, Melanie L. Schwandt, Vatsalya Vatsalya

**Affiliations:** ^1^ Department of Medicine, University of Louisville, Louisville, KY, United States; ^2^ Department of Medicine, Robley Rex VA Medical Center, Louisville, KY, United States; ^3^ Clinical Laboratory for Intervention Development of AUD and Organ Severity, Louisville, KY, United States; ^4^ School of Public Health and Information Sciences, University of Louisville, Louisville, KY, United States; ^5^ Department of Medicine, University of Louisville Alcohol Research Center, Louisville, KY, United States; ^6^ Division of Intramural Clinical and Biological Research (DICBR) National Institute on Alcohol Abuse and Alcoholism, Bethesda, MD, United States

**Keywords:** alcohol use disorder, alcohol-associated hepatitis, audit, IL-17, IL-22

## Abstract

**Introduction:**

Changes in the expression of cyto- and chemokines due to alcohol-associated liver disease (ALD) have been reported to be both protective and pathogenic. This study examined plasma levels of two key cytokines, Il-17 and Il-22, which construct the proinflammatory vs. anti-inflammatory axes across the spectrum of alcohol use disorder (AUD) and ALD including alcohol-associated hepatitis (AH) to determine the underlying status of the inflammation.

**Methods:**

Forty-two males and females aged 25-63 yrs. were grouped as healthy controls (HV[n=8]), AUD with no liver injury (AUDNLI [n=8]), AUD with liver injury (AUDLI [n=8]), non-severe alcohol-associated hepatitis (NSAH [n=9]), and severe alcohol-associated hepatitis (SAH [n=9]). Demographic, drinking, and clinical data were collected. Blood samples were collected at baseline (BL, all subjects) and during week 4 (W4, only patients) for IL-17 and IL-22; and statistically analyzed.

**Results:**

IL-17 was highly elevated in the SAH group both at BL and post-SOC. LTDH and BL IL-22 in non-severe AH patients were associated significantly. LTDH significantly predicted W4 IL-22 levels, positively (increasing) in NSAH and inversely (lowering) in SAH patients. BL and W4 IL-22 levels were significantly higher (4-fold, p≤0.001) in all AH patients compared to all AUD patients (AUROC=0.988, p≤0.001). IL-22 showed significant affinity with AST, AST: ALT ratio, total bilirubin, INR, and PT both at BL and W4. IL-22 was inversely associated with IL-1β; and positively with TNF-α and IL-8 both at BL, and W4. BL IL-17 showed a positive correlation with MELD (p=0.017) in all AH patients. In SAH, > 2-fold W4 IL-17 level compared to BL showed significant within subjects’ effects, p=0.006. In AUD patients without AH, the drop in IL-17 at W4 vs. BL showed a significant within subjects’ effect, p=0.031.

**Discussion:**

Drinking chronicity predicted opposite effects in IL-22 levels in NSAH (antiinflammatory) and SAH (pro-inflammatory) patients at post-SOC. BL IL-22 levels differentiated AH patients robustly from the AUD patients (with or without liver injury); and showed corresponding increases stepwise with the stages of ALD. IL-22 was closely associated with progression and injury markers of the liver; and response to the cytokines of pro-inflammatory nature. Pro-inflammatory indicator of IL-17 cell axis, IL-17 showed a strong positive association with MELD, a severity indicator of AH.

## Introduction

Alcohol-associated liver disease (ALD) refers to liver damage caused by the chronic excessive drinking of alcohol (1). ALD is often observed in patients who have pre-existing Alcohol Use Disorder (AUD). AUD is a mental disorder assigned by the Diagnostics and Statistics Manual (DSM) (Fifth Edition) and is characterized by compulsive excessive alcohol consumption over an extended time with associated loss of control and negative emotions (2). Alcohol consumption has been on the rise in the United States and worldwide; in 2019 there were 14.1 million adults in the United States that met the criteria for AUD (3). Interestingly, only 10-20% of chronic and heavy drinkers eventually progress to more severe and permanent forms of ALD, such as hepatitis and cirrhosis (4). There is likely a multitude of disease modifiers responsible for this progression and it still remains a challenge to identify this subset of AUD patients who will develop ALD (5). Such predictive ability could enable earlier interventions that may significantly reduce the proportion and likelihood of AUD patients who develop alcohol-associated hepatitis (AH).

Alcohol-associated hepatitis (AH) is an advanced and acute form of ALD that is attributed to elevated pro-inflammatory status in response to excessive alcohol intake and various other altered domains involved in liver injury (1). Severe alcohol-associated hepatitis (SAH) is characterized by a large systemic inflammatory response syndrome (SIRS) leading to severe liver inflammation and dysfunction with an associated high short-term mortality of 30-50% (6). Understanding the underlying cytokine cascading pathways holds the potential to predict the development and severity of ALD regardless of the changes indicated by the clinical measures. IL-17 and IL-22 are two particular cytokines involved in inflammation and cell death in ALD. While they have been implicated in alcohol associated liver disease, few studies have published on their role in AH (7–9).

Chronic and excessive alcohol intake causes gut dysfunction and increased gut permeability (10). This leads to the release of bacterial endotoxins, such as lipopolysaccharide (LPS), which bind to toll-like receptor 4 (TLR4) on the liver initiating a cascade of pro-and anti-inflammatory events (11–13). Alcohol metabolism in the liver further contributes to this inflammatory response through the production of acetaldehyde and concomitant reactive oxygen species (ROS) (14). This gut dysfunction and alcohol metabolism initiate a cascade of events in the liver involving the production of pro- and anti-inflammatory cytokines, including tumor necrosis factor-α (TNF-a), interleukin 1 (IL-1), interleukin 6 (IL-6), and transforming growth factor-beta (TGF-β) (15–18). These in turn lead to the differentiation of specific CD4^+^ T cells (namely, Th-17), which produce interleukin 17 (IL-17), interleukin 17F (IL-17F), interleukin 21 (IL-21), interleukin 22 (IL-22), and granulocyte-macrophage colony-stimulating factor (GM-CSF) (19–21). These in turn further promote neutrophil recruitment, inflammation, and fibrosis in ALD. This study evaluated the role of IL-17 and IL-22 as markers of inflammation in various stages of alcohol associated disease; spanning AUD without liver injury, AUD with liver injury (or Early Stage ALD), and ultimately non-severe and severe AH compared with the healthy controls.

## Materials and methods

### Patients and study paradigm

This study is one of the secondary aims of larger studies on ALD (both early-stage ALD and later stage such as hepatitis). The study on early-stage ALD participants was approved by the Institutional Review Board of the National Institute on Alcohol Abuse and Alcoholism at the National Institutes of Health, Bethesda, MD (under the screening protocol 98-AA-0009). This is indexed at the National Clinical Trial Website (www.clinicatrials.gov: NCT00001673). The AH and healthy volunteer (HV) samples used came from clinical trial NCT-01809132, which were approved by the IRB of the University of Louisville under protocol # 12.047. Subjects selected were those who were seeking study-based treatment for alcohol detoxification as described in the clinical protocol. This included admission at an NIAAA inpatient facility for 28 days, alcohol detoxification, and standard of care (SOC) treatment. All eligible patients were consented prior to being enrolled in the trial.

In the above-mentioned large studies, all patients had to be diagnosed with AUD based on DSM-IV criteria prior to being eligible for enrollment (2). Primary exclusion criteria included: diagnosis of severe psychiatric disease, diagnosis of severe somatic illnesses such as decompensated cardiovascular disease, advanced lung disease, or renal failure, positive HIV test, pregnancy or ongoing breastfeeding, and/or positive urine drug screen. These patients were then allowed to decide during their first three days of admission to be a part of the SOC group or to be eligible for randomization in the overarching studies. SOC entailed obtaining thorough history and physical examinations, neurologic evaluations, laboratory tests, proper nutrition, discharge planning and referrals for treatment of AUD (as needed), and prescribing diazepam for severe withdrawal symptoms per need. In addition, SOC study participants received either acamprosate or a placebo as part of the NIH study. Acamprosate and diazepam are not known to have any effect on liver function, these patients were included in this secondary study (22, 23). Further details on admission, inclusion and exclusion criteria, and inpatient treatment can be found in several of our other studies (24–27).

Forty-two participants, both male and female, aged 25-63 years with AUD met inclusion criteria for the study. These patients were separated into 5 groups: healthy controls (HV[n=8]), alcohol use disorder (AUD) with no liver injury (AUDNI [n=8]), AUD with liver injury (AUDLI [n=8]) also identified as early-stage ALD, non-severe AH (NSAH [n=9]), and severe AH (SAH [n=9]). An alanine aminotransferase (ALT) measure of greater than 40 IU/L was used to indicate liver injury in AUD since ALT of 40 IU/L is the upper limit of normal and values greater than 40 IU/L indicate early stage liver injury (28). Aspartate transaminase (AST) greater than 34 IU/L was considered clinically significant (based on Medline Plus guidelines till 2014 yr. when the samples were collected). An AST : ALT ratio of >1.5 is considered indicative of AH (29). Clinical data and blood samples were collected at baseline (BL, all subjects) and week 4 (W4, patients who remained admitted, and while following 3-4 weeks of standard of care medical management including detoxification and counseling). Severe AH was defined as having a MELD score greater than or equal to 20. These AH patients received a treatment of 28-day steroid course. Baseline drinking assessments were collected using the following questionnaires: Timeline Followback (TLFB) questionnaire, which assesses Total Drinks in the past 90 Days (TD90), Number of Non-Drinking Days in the past 90 days (NNDD90), Average Drinking Days (AveDD90) and Heavy Drinking Days in the past 90 Days (HDD90) (29, 30); Lifetime-Drinking History [LTDH (31)]; and Alcohol Use Disorders Identification Test (AUDIT) (32). The endpoint for this study is 4 weeks. Week 4 is an endpoint because it encompassed average inpatient treatment and steroid course. Inpatient care duration for AUD is 3-4 weeks and additionally, steroid treatment for alcoholic hepatitis is 28 days or more.

### Laboratory assays

The collected serum samples underwent laboratory analyses, which included collection of the following: complete metabolic panel (CMP), complete blood count (CBC), inflammatory markers (c reactive protein [CRP], immunoglobulins A [IgA], G [IgG], and M [IgM]), and cytokine assays. ELISA was used to assess plasma levels of the cytokine IL-22 (BMS2047, Invitrogen) and high-sensitivity ELISA was used to measure plasma IL-17 (BMS2017HS, Invitrogen) per manufacturer’s instructions; plasma was diluted 1:2 for both respective assays. Results were read on a Spectra Max Plus 384 plate reader and analyzed using SoftMax Pro software (Molecular Devices, San Jose, CA). Other cytokines mentioned were measured using Luminex platforms.

### Statistical analysis

Demographics (Age, Sex, Body Mass Index (BMI) and drinking history) were collected and used in the analysis as factors and covariates (**Table 1**). Differences in the demographic characteristics, drinking history measures, and liver injury markers were evaluated using univariate factorial ANOVA (from two-group) and one-way ANOVA across multiple group analyses. Repeated ANOVA was performed to test the differences at the study end from baseline assessment. Linear regression model was used for identifying the effect and power of the association. SPSS 27.0 (IBM, Chicago, IL), Microsoft 365 2020 version (MS Corp, Redmond, WA), and GraphPad Prism (GraphPad Software, San Diego, CA) were used for statistical analyses. Statistical significance was set at p ≤ 0.05. Data presented as Mean ± Standard Deviation (M ± SD) unless otherwise specified in Figure/s or Table/s.

**Table 1:** Demographic, drinking and clinical measures.

Groups	HEALTHY VOLUNTEERS		AUD (NO INJURY)		AUD (INJURY)		AAH (NON-SEVERE)		AAH (SEVERE)	
Measures	n=8		n=8		n=8		n=9		n=9	
BL	W4	BL	W4	BL	W4	BL	W4	BL	W4
**AGE (Years)**	28.4 (4.4)	–	40.3 (13.5)	–	43.6 (7.5)	–	46.0 (11.5)	–	48.8 (8.7)	–
**BMI (kg/m^3^)**	24.7 (3.3)	–	26.8 (7.0)	–	27.5 (5.5)	–	30.0 (10.1)	–	36.2 (7.4)	–
**TFLB (Unit Score)**	1.4 (0.5)	–	15.5 (5.5)	–	17.4 (5.5)	–	–	–	–	–
**AUDIT (Unit Score)**	–	–	–	–	–	–	24.7 (6.0)	–	20.3 (8.7)	–
**LTDH (Years)**	9.71 (5.6)	–	11.0 (5.6)	–	17.0 (9.4)	–	17.8 (14.4)	–	24.8 (16.7)	–
**TBIL (U/I)**	0.5 (0.1)	–	0.6 (0.2)	0.5 (0.2)	0.4 (0.3)	0.4 (0.2)	12.9 (10.9)	2.4 (1.2)	21.7 (8.8)	11.8 (9.6)
**TP (g/dl)**	–	–	7.1 (0.4)	6.3 (0.5)	7.4 (0.6)	6.8 (0.6)	5.7 (1.2)	7.3 (0.8)	5.8 (0.8)	6.3 (1.2)
**ALB (g/dl)**	4.1 (0.3)	–	4.1 (0.2)	3.6 (0.3)	4.3 (0.4)	3.8 (0.3)	2.5 (0.5)	3.7 (1.0)	2.3 (0.3)	2.8 (0.4)
**AST (U/I)**	24.4 (2.9)	–	29.0 (13.4)	26.4 (12.5)	59.3 (42.4)	25.6 (10.8)	183.6 (86.3)	53.5 (22.5)	137.4 (52.1)	63.6 (22.8)
**ALT (U/I)**	20.4 (5.2)	–	25.6 (8.1)	39.6 (19.7)	70.1 (28.0)	51.56 (29.5)	90.4 (45.1)	35.3 (16.8)	54.0 (30.3)	44.4 (22.2)
**AST : ALT Ratio**	1.2 (0.3)	–	1.1 (0.4)	0.8 (0.3)	0.8 (0.4)	05 (0.6)	2.2 (0.9)	1.6 (0.6)	3.0 (0.9)	1.8 (1.2)
**MELD (Unit Score)**	–	–	–	–	–	–	17.3 (2.1)	11.6 (3.2)	24.0 (3.5)	22.6 (6.9)

AUD, alcohol use disorder; AH, alcohol associated hepatitis; BMI, body mass index; TLFB, Timeline Followback; AUDIT, Alcohol Use Disorders Identification Test; LTDH, lifetime drinking history; ALB, albumin; AST, aspartate aminotransferase; ALT, alanine transaminase; AST, aspartate aminotransferase; ALT ratio, ALD progression indicator; TBIL, total bilirubin; TP, total protein; MELD, Model for End-Stage Liver Disease. Units in parentheses beside the measures. Standard deviation along with the means. Across all the group significant difference. HV vs. AUD no injury. HV vs. AUD with early-stage liver injury. HV vs. non-severe AAH. HV vs. severe AH. AUD with no liver injury vs. AUD with early-stage liver injury. AUD with no liver injury vs. non-severe AAH. AUD with no liver injury vs. severe AAH. AUD with early-stage liver injury vs. non-severe AAH. AUD with early-stage liver injury vs. severe AAH. non-severe AAH vs. severe AAH. Data presented as Mean ± Standard Deviation (M ± SD).

## Results

### Demographics and clinical presentation

The HV group were younger with an average age of 28.4 ± 4.4 and had an average BMI below 25 compared to the AUDNI, AUDLI, NSAH and SAH groups. The participants in the AUD with no injury and AUD with early-stage liver injury were younger on average than the non-severe (NSAH) and severe AH (SAH) groups. The TFLB score for the AUDNLI was slightly lower than the AUDLI. The AUDIT scores for the NSAH group were higher compared to the SAH group. HV had less LTDH than all the groups and SAH had the highest LTDH at 24.8 ± 16.7 years. Total bilirubin increased across all five groups with the highest levels found in the SAH. The total bilirubin levels decreased more from BL to W4 in the NSAH compared to the SAH group.

### Presentation of plasma IL-17 AND IL-22

**Figure 1:**
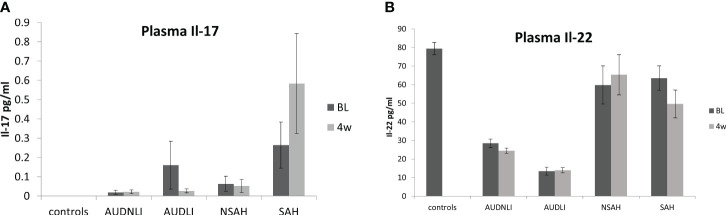
levels of **(A)** IL-17 and **(B)** IL-22 in human plasma (+/- SEM) at baseline (dark bars) and week 4 (lighter bars). Groups were as follows: AUDNLI, AUD no liver injury; AUDLI, AUD with liver injury; NSAH, non-severe alcohol associated hepatitis; SAH, severe alcohol associated hepatitis. IL-17 tended to increase in SAH. IL-22 decreased in AUD from healthy volunteers but increased in SAH. There was no statistical difference between time points within any group. Statistical significance was set as p< 0.05.

**Figure 2:**
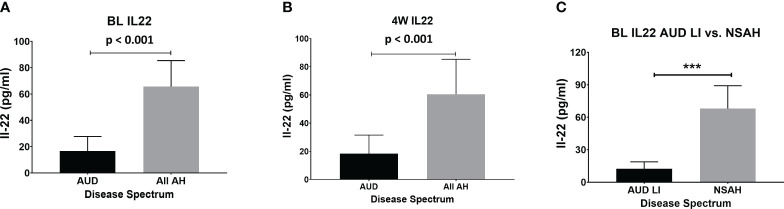
IL-22 increased significantly in all AH (combined severe and non-severe) compared to all AUD (combined with and without liver injury) subjects at **(A)** baseline (p=0.001) and **(B)** week 4. **(C)** Baseline IL-22 increased significantly in non-severe AH compared to AUD with liver injury (p=0.001). Statistical significance was set as p< 0.05.

IL-22 was significantly lower in HV compared to the AUD; however, it was elevated in both AH groups at the intake presentation (**Figure 1**). Notably, BL IL-22 was significantly higher (4-fold, p ≤ 0.001) in all AH patients compared to all AUD patients (AUROC=0.988, p ≤ 0.001) (**Figure 2A**). This difference remained statistically significant and correspondingly similar at W4 (**Figure 2B**). We also found that the BL IL-22 levels were significantly higher in NSAH compared to AUDLI, p ≤ 0.001 (**Figure 2C**). There was huge numerical difference between AUDNLI and AUDLI, albeit no statistically significant difference in the BL IL-22 levels; and similarly, between NSAH and SAH. We found the same response in the W4 IL-22. (**Figure 1**). Plasma IL-17 was at the highest levels in SAH; however, this elevation was not statistically significant different largely due to the internal variability in data within each group.

### LTDH correlates with IL-22 IN alcohol associated liver disease

**Figure 3:**
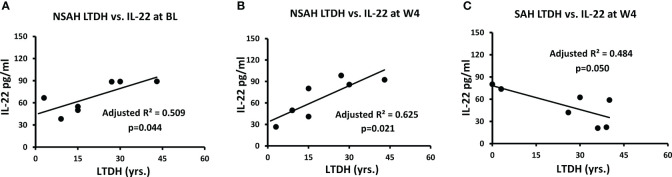
Correlation of IL-22 plasma levels with LTDH in non-severe alcohol associated hepatitis at **(A)** baseline (p=0.044) and **(B)** week 4 (p=0.021), and **(C)** in severe alcohol associated hepatitis at week 4 (p=0.05). Statistical significance was set as p< 0.05.

All of AH patients together reported 1.5 times higher LTDH (21.1 ± 15.40 vs. 13.8 ± 7.95) compared to all AUD patients together. In NSAH, both BL and W4 IL-22 were significantly associated with LTDH (p=0.044 and p=0.021 respectively) (**Figures 3A, B**). In SAH, LTDH was significantly associated only with W4 IL-22, p=0.050 (**Figure 3C**). No association was found with LTDH and IL-17 in SAH subjects at baseline or at the study end.

### Paradox in the IL-17 responses observed in AH and AUD patients

**Figure 4:**
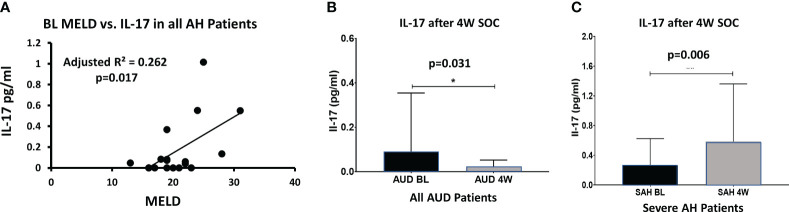
**(A)** At baseline, IL-1IL-17 levels correlate with MELD score (p=0.017) in all AH patients (with-in subject). **(B)** In all AUD patients, IL-1IL-17 decreased significantly from BL to week 4 with standard treatment (p=0.031, within-subject difference). **(C)** However, in severe AH patients, IL-1IL-17 increased significantly during the 4 weeks of standard care (p=0.006, within-subject difference) suggesting unresolved inflammatory activity (and/or a feedback loop). Statistical significance was set as p< 0.05.

BL IL-17 was significantly and positively associated (adjusted R²=0.262, p=0.017) with MELD when both groups of AH patients were considered together (**Figure 4A**); this association lost its significance at W4, with increased IL-17 and an increase in variance of IL-17 (**Figure 4C**). There was more than a 2-fold increase at W4 IL-17 compared to BL values in SAH which showed significance within subjects’ effects, p=0.006, suggesting unresolved and growing pro-inflammatory activity (**Figure 4C**). On the other hand, in the AUD patients, the drop in W4 IL-17 level from baseline showed significance within subjects’ effect, p=0.031, supporting efficacy of medical management (**Figure 4B**). In AUD patients, there was a significant 73% decrease in IL-17 from BL to W4 after SOC. In contrast, in the AH patients IL-17 remained significantly higher (by 2-folds) at W4 compared to the BL even after the SOC. Notably, W4 IL-17 was observed at a highly elevated level, and statistically significant in all the AH patients in comparison to all the AUD patients as well (p=0.031, data not presented figuratively).

### Association of Th-cell axis cytokines with liver injury, and candidate pro-inflammatory cytokines in AUD patients with or without ALD

We performed a panorama analysis for all the AUD patients (with or without ALD). BL IL-17 showed specific and unique predictive significance for AST values at W4 (**Table 2**) at moderate effects. On the other hand, IL-22 showed a wide range of significant associations with several liver injury markers both at baseline as well post-SOC (either at the higher end of moderate or high effects). AST (Aspartate Transaminases), AST: ALT (Alanine transaminases) ratio, Total Bilirubin (Bili-Tot), INR (International Normalized Ratio) and PT (Prothrombin Time) were positively associated with IL-22 both at baseline and post-SOC (**Table 2**). We did not find any significant traits in ALT values (data not presented).

**Table 2:** Association of Th-cell axis cytokines with candidate liver injury markers, and candidate pro-inflammatory cytokines.

Correlation Matrix								
SPEARMAN’S R					P-VALUE			
	**IL-17 BL**	**IL-17 W4**	**IL-22 BL**	**IL-22 W4**	**IL-17 BL**	**IL-17 W4**	**IL-22 BL**	**IL-22 W4**
**AST BL**	0.307	0.067	0.348	0.522	0.05739	0.70558	0.03453	0.00218
**AST W4**	0.395	0.073	0.684	0.648	0.04162	0.71739	0.00016	0.00046
**AST : ALT BL**	0.267	0.118	0.623	0.697	0.10059	0.50518	3.77936E-05	9.34227E-06
**AST : ALT W4**	0.229	-0.136	0.732	0.671	0.25090	0.50010	0.00003	0.00024
**Bil-Tot BL**	0.293	0.198	0.464	0.581	0.07019	0.26171	0.00380	0.00049
**Bil-Tot W4**	0.136	0.031	0.776	0.685	0.49855	0.87829	0.00001	0.00016
**INR BL**	0.172	0.118	0.756	0.696	0.33881	0.51393	0.00000	0.00001
**PT BL**	0.081	0.206	0.592	0.55	0.64975	0.24233	0.00036	0.00112
**IL-22 BL**	-0.067	0.137	NA	0.861	0.68210	0.45325	NA	0.00000
**IL-22 W4**	0.15	0.041	NA	NA	0.41179	0.82460	NA	NA
**IL-1 BL**	0.035	-0.102	-0.529	-0.41	0.82756	0.57215	0.00044	0.01973
**TNF- BL**	0.382	0.287	0.359	0.66	0.01381	0.10575	0.02275	0.00004
**IL-8 BL**	0.287	0.177	0.438	0.718	0.06860	0.32380	0.00468	0.00000
**P-Value**		**SPEARMAN’S R**
<=0.05	<=-0.5	Moderate negative
<=0.05	<=-0.7	Strong negative
<=0.05	<=-0.5	Moderate positive
<=0.05	<=-0.7	Strong negative

AST, Aspartate Transaminase; AST : ALT, AST by ALT (Alanine Transaminase) ratio; Bil-Tot, Bilirubin Total; INR, International Normalized Ratio; PT, Prothrombin Time; IL-22, Interleukin 22; IL-17, Interleukin 17; IL-1b, Interleukin 1b; TNF-α, Tumor Necrosis Factor alpha; IL-8, Interleukin 8. NA, Not applicable. Lightest Grey = Moderate Negative; Lighter Grey = Strong Negative; Dark Grey = Moderate Positive; Black = Strong Positive.

Similarly, the association of candidate Th-cell cytokines, IL-17 and IL-22 were uniquely expressed in the context of other pro-inflammatory cytokines. BL IL-17 and BL TNF-α showed moderate effects of significant association (**Table 2**). IL-22 on the other hand, showed significant positive association with TNF-α (moderate effects), and IL-8 (BL: Moderate, and Post-SOC: Strong). With IL-1β, we found that IL-22 showed negative moderate effects of significant association (**Table 2**).

## Discussion

The functional aspects of so-called pro- and anti-inflammatory cytokines are so interwoven, that it is often difficult to separate a particular cytokine into one or the other of these two classifications. Such is the case with IL-17 and IL-22 cytokines that mostly originate from Th-17 cells. In general, IL-22 is considered beneficial, and IL-17 as detrimental in any inflammatory pathology. We see such activity from our findings also in alcohol-associated liver disease. In this study, we found that at baseline, IL-22 levels significantly differentiated AUD from AH patients whereas IL-17 tended to increase in conjunction with liver injury. Several other studies have found definite increases in IL-17 with ALD. For example, Lemmers et al. found that IL-17 plasma levels increased in patients with alcoholic liver disease (including AH) compared with healthy subjects (7). A 2013 study of 21 AH patients with time points at BL, 14 days (W2) and a month (W4) measured IL-17 and IL-22 in plasma using ELISA (9). They found that IL-17 was 1.5 times more elevated in AH compared to healthy controls, which is consistent with the data in this study. There were several other similarities found with this current study. AH IL-17 increased, and IL-22 decreased at W4 compared to BL, although these differences were not statistically different. In addition, like this study, their AH IL-22 data did not differ from healthy controls. Another group studied IL-22 levels in alcohol associated liver disease and found that they correlated with MELD (8). While we did not see a similarly strong positive correlation of IL-22 with MELD, Parfieniuk-Kowerda’s findings are consistent with our findings of positive relationships with other markers of liver damage.

**Figure 5:**
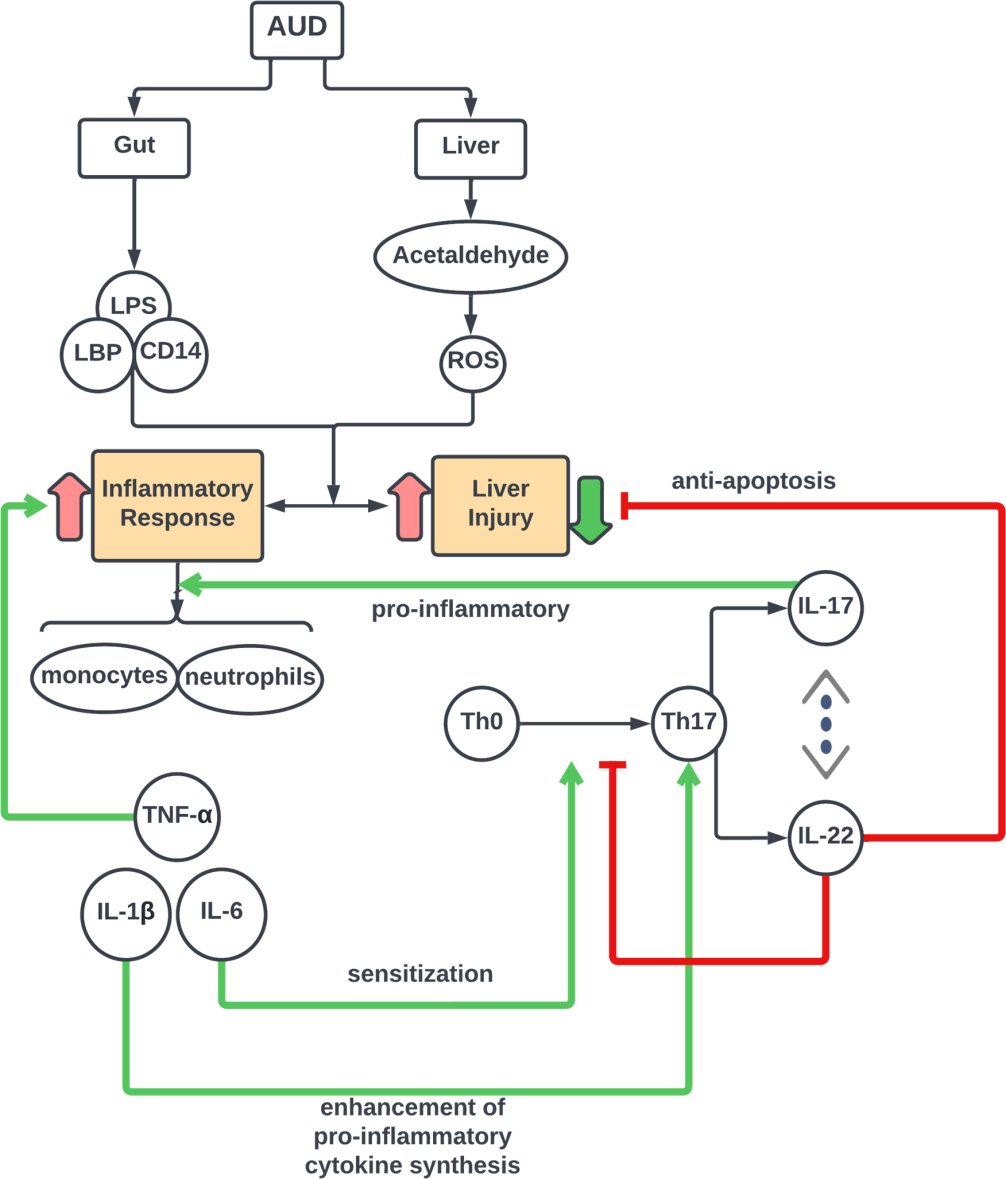
Schema of IL-17 Cell Axis Response and interaction of cytokine pathways in Alcohol-associated Liver Disease. Model for Th-cell associated pathophysiology of alcohol–induced gut dysfunction and pro-inflammatory response leading to liver injury in acute alcohol associated hepatitis.

BL IL-22 correlated negatively with BL IL-1β. Since IL-1β is a key cytokine in the pro-inflammatory response, this was the only correlation to directly support the anti-inflammatory nature of IL-22. It can be surmised that the other IL-22 correlations are indicating a response to liver injury, and/or increased IL-22 resistance (**Figure 5**).

Lu et al. (2017) used human samples from the TREAT consortium to measure IL-17 and IL-22 in the serum of healthy volunteers, heavy drinkers, and AH subjects using a multiplex protein assay. Unlike our study, they found that AH subjects had an increased IL-22 compared to healthy controls. However, similarly to our study, they found IL-22 increased from heavy drinkers to AH. They found no significant differences in IL-17. The authors thought perhaps increased IL-22 resistance was the reason for increased IL-22 in AH subjects. Other studies have shown increases in the cytokines and growth factors that induce IL-22 with increasing injury. Accordingly, since levels of all these IL-22 inducers are increasing, it is reasonable to expect that IL-22 will also increase. It is possible that this is the result of an overwhelming inflammatory response that increases cytokines, such as IL-6, IL-1β, and IL-23 that in turn signal Th-17 to make increased IL-22 (**Figure 5**). There is some pivot, however, where the levels of the pro-inflammatory inducing cytokines will begin to decrease, and IL-22 will be able to employ its beneficial effects.

Of these two cytokine measures, IL-22 is easier to detect using standard protein assays due to high levels of expression when triggered. And accordingly, statistically significant relationships are more clearly measurable. BL versus W4 IL-22 measures correlate well, so at least in this study, it consistently increases over the spectrum of liver injury and post-treatment. It correlates negatively with IL-1β (a further downstream pro-inflammatory cytokine), correlates positively with liver damage markers (AST: ALT, AST) as well as shows corresponding initiation of pro-inflammatory cytokines such as TNF-α and IL-8 in our findings.

Additionally, the number of years of alcohol drinking was found to be closely associated with IL-22 in non-severe AH patients and was predictive of W4 IL-22 levels in these same subjects, indicating that increased drinking is associated with increases in IL-22. In turn, as liver injury increases, levels of IL-22 will also increase in an attempt to mitigate the inflammation and initiate regenerative processes (33). Stoy et al. also measured IL-22 expressing T cells using FACs and found that patients with a favorable short-term course, as measured by the Glasgow score, had higher levels of these IL-22 producing Th17 cells (9). Thus, the overall notion is that, even though IL-22 is apparently increasing with markers of liver injury, the ultimate outcome of increased IL-22 may be liver regeneration and survival, although our study did not include these endpoints per se.

In this study, as anticipated in the Th-17 cell response, IL-17 increased overall with liver injury in a step-wise progression per the corresponding increase in liver severity. Whereas plasma IL-22 decreased in healthy volunteers and in AUD, albeit increased again in AH, this observation is important. This response could reflect a subtlety in signaling that occurs during alcohol associated inflammation. The decrease in IL-22 in AUD could be a result of increased production of TGF-β by Kupffer cells in the initial inflammatory response to alcohol overconsumption; TGF-β is known to decrease IL-22 production (34). Kim et al. found that serum TGF-β levels increased in alcohol-dependent subjects compared to healthy controls (35). Increasing concentrations of TGF-β were found to inhibit IL-6-induced production of IL-22 in Th17 cells in a model of autoimmune disease (34). Either IL-23 or IL-6 could stimulate IL-22 production independently of each other. Another study has shown liver IL-6 to decrease, though minimally, in AH (36). The results in our study were consistent with other studies that found blood IL-6 increased with increasing severity of alcoholic liver disease (37, 38). It is possible that the lower levels of IL-22 found in AUD patients could have been due to similar TGF-β signaling as a part of the early inflammatory response. Recently, it was found that both Th17 cells and IL-23R expression are increased in patients with AH and Hepatitis B, respectively (39). An increase in IL-23 activity, possibly independent of IL-6, may contribute to the increase in IL-22 seen in AH patients as compared to AUD subjects. Measurement of plasma IL-23 in AUD and AH patients would be helpful in supporting this hypothesis, which could be a next direction of investigation. Additionally, measurement of suppressor of cytokine signaling 3 (SOCS-3) in AUD and AH might be informative, since it is thought to be involved in a negative feed-back loop for IL-22 production to enable signal transducer and activator of transcription (STAT3) signaling (40). Evidence has shown that STAT3 signaling is indeed deficient in early alcohol associated liver disease (41). It may be that the lower levels of IL-22 in AUD would correlate with increased SOCS-3 activity.

IL-22BP is the endogenous decoy ligand for the IL-22/IL-10 dimer. Its lower levels in AH are associated with increased mortality (42). IL-22 was found to have a feedback loop that reduces IL-22R, thus decreasing IL-22 bioactivity. IL-22BP is thought to negate the IL-22 suppression of IL-22R. This could be one of the reasons a high association of IL-22 levels with liver damage markers is seen. There is also a line of thought in the literature that IL-22 is detrimental in liver disease (43, 44). Recent literature has found, in opposing results, that IL-22 is both beneficial (45) in a mouse model but not beneficial (46) in clinical studies of acute on chronic liver failure, which AH is a subset of. However, use of a mouse model for studies on IL-22/IL-22BP is lacking because humans produce several thousand-fold more IL-22BP than mice do (45, 46) and humans produce three IL-22BP isoforms (47) that mice do not. This signaling system is so complex that it is difficult to draw strong conclusions from *in vitro* and mice experiments.

Currently, the progression from AUD to AH is not well understood due to a lack of comprehensive study of the evolution of inflammation across the spectrum of AUD and ALD. An understanding of these mechanisms could lead to earlier intervention and hopefully prevention of devastating liver disease and its sequelae. We found a substantial association of IL-22 and AST, as well as AST : ALT ratio (both are markers of liver injury progression) both at baseline and post-SOC in the spectrum of ALD, supporting the role of IL-22 in the progression of ALD. IL-17 binds to the IL-17A/C receptors, which are ubiquitously expressed, but are also induced by stress on hematopoietic cells and fibroblasts. The IL-17 receptors most critical in ALD are most likely those expressed on stellate cells (Lemmers et al., 2009). The signal is propagated by transcription factor STAT3 (Arab et al., 2019; Qu et al., 2013). Upon stimulation with IL-6, IL-23, and IL-1β through the AHR receptor Th-17 cells will also release IL-22. IL-22 then binds to the heterodimeric IL-10R2/IL-22R1. IL-10R2 is ubiquitously expressed, but expression of IL-22R1 receptors is largely limited to epithelial cells, which in the case of the liver are hepatocytes. IL-22R1 in the liver is also found on hepatic stellate cells, liver progenitor cells, and some fibroblasts. IL-22 signaling is involved in tissue repair and antimicrobial activity. It can also inhibit apoptosis and induce proliferation through STAT3 signaling (Kong, Feng, Mathews, & Gao, 2013; Kong et al., 2012; Qu et al., 2013).

Recent studies point to the role of hepatic progenitor cells (HPC) in AH (36, 48). It was found that HPC accumulate in AH patients, but they fail to differentiate into hepatocytes. This group found a decrease in STAT3 and pSTAT3 in AH livers and thought that this might be a contributing factor to the lack of regeneration seen in AH. So even while IL-22 might be increasing in AH, the most important downstream activity of the transcription factor STAT-3 was decreasing. This points to dysfunctional IL-22 signaling in AH by increased IL-22 resistance. IL-22 was shown to inhibit epidermal differentiation of keratinocytes (Boniface 2005), but its effect on differentiation of HPC to hepatocytes is not known. Changes in levels of IL-22, which is known to be associated with liver regeneration (33) could be a part of the dysfunctional regenerative response that seems to occur in AH.

The protective effect of IL-22 on the liver was first reported in 2004 (49, 50). Clinical trials have been undertaken for an IL-22 conjugate for treatment of AH. The Phase 2A trial showed benefits with no adverse events (51). Our data suggests that this treatment might be especially beneficial during AUD, when IL-22 levels are lower, to increase their levels and perhaps offset progression to AH. The treatment results in extremely high levels of IL-22 (~700,000 pg/ml after IL-22Fc compared to 200 pg/ml before in healthy volunteers). Therefore, in addition to interplay with IL-22BP and receptors, there is no doubt a dose-dependent effect for IL-22, that could be a potential medical management modality. This may explain why this treatment works and why we found relatively lower IL-22 levels (60 pg/ml) in AH correlating with liver damage and dysfunction markers.

### Limitation

This study has several limitations. This is a clinical natural history investigation that was developed as a pilot study to test some of the pathways emerging in recent literature specially the findings on IL-22 and Il-17 on liver damage. Thus, the design of the study was built as high risk, high yield; therefor, including more subjects in such a study concept is not possible once the study has ended. Additionally, age-matching was not used in the study because it was open for anyone above 21 years of age with AUD. We narrowed the criteria to a range of 25-63 years old. Because we did not know what patients would enroll, it limited us from age-matching the different groups.

The study subject number is small for an ELISA based discovery project due to inherent variability in the technique. The scope for identifying sex-differences was not anticipated in this study due to small sample size,; however, we attempted to test i statistical trends to assess if they existed specifically for this purpose. ALD patients received medical management based on the standard of care protocol, that might vary based on their clinical indications over the period the assessment. For example, patients with AUD were admitted for inpatient detox and those who scored high enough using the Maddrey’s Discriminant Factor algorithm for alcoholic hepatitis received a course of steroids. Even though the severity by category of AH may have equated such ALD in one group, their demographics (modifiers), progression (injury and inflammation) may have varied and that could have attributed to a differing level of cytokines at the end of study. We did not run Peth since the drinking history was collected for 3 months using Timeline followback. Finally, we were unable to find between group differences as they related to IL-17 due to peripheral cytokine production acting as a confounder.

## Conclusions

The extensive range of AUD and alcohol associated liver disease investigated in this study showed that IL-22 levels correlated positively with markers of liver injury. Number of years of alcohol drinking was closely associated with IL-22 in non-severe AH patients and predicted W4 IL-22 levels. The effects of IL-22 are predominantly anti-inflammatory in nature and increased IL-22 production is thought to be in response to liver injury and/or IL-22 resistance. At baseline, IL-22 levels significantly differentiated AH patients from AUD patients; regression analysis showed that IL-22 predicted progression from AUD with liver injury to moderate AH. Overall, in the alcohol associated groups, IL-22 at baseline correlated very strongly with AST: ALT ratio at week 4. It seems that the compensatory mechanism of IL-22 is superseding the IL-17 response when ALD is not severe. This could be attributed to the IL-22’s survival factor response in liver (50). In severe AH patients, IL-17 response is more meaningful and correlated with the severity assessment. IL-17 response shows that the pro-inflammatory pathway is well underway in severe AH; and IL-22’s compensatory response could have receded. The accuracy of these predictions will only be verified by large long-term longitudinal studies that would begin by enrolling AUD patients, and then following them through potential progression to liver injury over a long period of time. The balance of these two cytokines produced by the same cell may be the key to predicting progression from AUD to severe liver injury.

## Data availability statement

The original contributions presented in the study are included in the article/supplementary materials. Further inquiries can be directed to the corresponding author.

## Ethics statement

The studies involving humans were approved by University of Louisville IRB, and NIAAA CNS IRB. The studies were conducted in accordance with the local legislation and institutional requirements. The participants provided their written informed consent to participate in this study.

## Author contributions

VV is the project PI and developed the study design. JF, MS, MLS, DJ and VV performed the sample handling and analyses. VV, MS, JF, DJ, HH, MLS and MK performed the data analysis. JF, MS, MK, AR, DJ, RB and VV interpreted the data analysis. JF, MS, DJ, AR, RB and VV wrote the manuscript. VV, MK, MLS and AR reviewed and scientifically contributed to the manuscript. All authors contributed to the article and approved the submitted version.
